# Antinociceptive effects of subcutaneous butorphanol administration in healthy horses

**DOI:** 10.3389/fvets.2026.1970768

**Published:** 2026-10-09

**Authors:** T. M. Masters, C. De Gennaro, S. L. Messer, J. L. Khatib, R. A. Moura, M. F. Mallicote, L. Chiavaccini

**Affiliations:** 1 Department of Comparative, Diagnostic and Population Medicine, College of Veterinary Medicine, University of Florida, Gainesville, FL, United States; 2 Department of Large Animal Clinical Sciences, College of Veterinary Medicine, University of Florida, Gainesville, FL, United States

**Keywords:** butorphanol, equine, pain pressure threshold, quantitative sensory testing, subcutaneous injection, thermal threshold

## Abstract

**Introduction:**

Subcutaneous (SQ) butorphanol administration maintains target plasma concentrations in horses longer than intravenous administration with fewer adverse effects. This study aimed to evaluate whether SQ butorphanol also produces clinically relevant antinociception in healthy horses.

**Methods:**

In a blinded, randomized, cross-over study, eight horses received 0.1 mg/kg butorphanol or equal volume (0.01 mL/kg) saline SQ in the cervical region. Thermal (TT) and pain pressure (PPT) thresholds at different body locations, along with physiological and behavioral variables, were assessed at baseline and up to 480 min post-injection. Data were analyzed using Stata/BE 17.0 and summarized as mean ± SD or median [interquartile range (IQR)], as appropriate. Mixed-effects linear regression (horse random effect) assessed treatment, time, and interaction effects (*p* ≤ 0.05). Adjusted marginal means (95% CI) were derived post-estimation.

**Results:**

Butorphanol produced mild, transient sedation (Wald *χ*^2^ = 145.5, *p* < 0.01) with a significant treatment × time interaction (LR *χ*^2^ = 46, *p* < 0.01), peaking at 45–90 min after injection. Heart rate decreased over time with both treatments, and respiratory rate was briefly reduced at 10 min with butorphanol. Mean arterial pressure was unchanged overall, and no hypotension occurred. TT and thermal excursion (TE%) showed significant treatment × time interactions (TT: LR *χ*^2^ = 18.1, *p* = 0.02; TE%: LR *χ*^2^ = 18.4, *p* = 0.02) at the coronary band only, decreasing 60–240 min post-butorphanol injection. PPT showed no treatment × time interaction at any site.

**Discussion:**

No clinically relevant treatment effect on TT or TE% was detected. Pressure pain threshold increased modestly for 1–2 h, but it was not statistically significant, likely due to variability and limited power. Subcutaneous butorphanol at 0.1 mg/kg produced mild, transient sedation with clinically acceptable cardiopulmonary effects, but without consistent antinociception.

## 1 Introduction

Equine pain management lags behind its small animal counterpart largely because horses suffer unique, life-threatening side effects from standard analgesic drugs (1). Non-steroidal anti-inflammatory drugs (NSAIDs) remain the mainstay of analgesic therapy in horses; however, their use is associated with potentially serious adverse effects including right dorsal colitis, gastroduodenal ulceration, and renal papillary necrosis (2). Opioid analgesia remains underutilized in equine medicine despite its well-documented efficacy in pain management, largely owing to concerns about undesirable side effects (1, 3). In particular, full mu-opioid receptor agonists are particularly avoided because they may induce excitatory behaviors and impair gastrointestinal motility, both of which can be particularly problematic in horses with underlying disease or compromised gastrointestinal function (4, 5).

Butorphanol, a κ-opioid receptor agonist and μ-opioid receptor antagonist, is widely used in equine practice due to its sedative and analgesic properties, favorable safety profile, and accessibility (1). It is most commonly administered intravenously (IV) or intramuscularly (IM). Butorphanol is often administered along with an α_2_-adrenoreceptor agonist to enhance sedation quality (1). While the drug is approved for IV administration at 0.1 mg/kg every 3–4 h, much lower doses (0.01–0.02 mg/kg) are typically used for sedation (6). Despite its widespread use, the antinociceptive efficacy of butorphanol in horses remains poorly substantiated. Previous research suggests that butorphanol may be more efficacious for visceral than somatic pain. However, much of this work was performed in healthy horses with experimentally induced colic (5, 7). A single study reported increased thermal nociceptive thresholds (TT) in foals when 0.1 mg/kg was administered IV (8), but robust evidence of meaningful somatic or visceral antinociception at clinically relevant doses in adult horses remains lacking.

Identifying administration routes that optimize butorphanol’s analgesic efficacy with increased dosages, while mitigating adverse effects has therefore remained a clinical priority in equine pain management. Intramuscular butorphanol administration does not meaningfully reduce the adverse effects associated with IV administration (9). In contrast, subcutaneous (SQ) injection has demonstrated favorable pharmacokinetics, with an estimated bioavailability of 87% in horses (4). At 0.1 mg/kg SQ injection, butorphanol achieves target plasma concentrations of approximately 10 ng/mL, and maintains them longer than equivalent IV dosing, while producing fewer physiological and behavioral side effects (4). Following these pharmacokinetic findings, SQ butorphanol has been increasingly adopted in equine practice with the intent of providing prolonged analgesia, commonly used for horses affected by acute abdomen (colic). Its use has been anecdotally recommended for analgesia in colic cases, including prior to transport to a referral hospital, underscoring both the clinical relevance of this route and the urgency of establishing its antinociceptive efficacy. In fact, the pharmacokinetic profile described by Chiavaccini et al. (4) did not include assessment of antinociceptive endpoints. Therefore, it remains unclear whether the plasma concentrations achieved following subcutaneous administration of 0.1 mg/kg butorphanol translate into clinically meaningful antinociceptive effects.

Hence, the primary objective of this study was to determine whether SQ butorphanol produces clinically significant antinociceptive effects in horses. It was hypothesized that 0.1 mg/kg SQ butorphanol would increase nociceptive thresholds for at least 2 h after administration, without clinically relevant changes in vital parameters or behavior.

## 2 Materials and methods

### 2.1 Ethical statement

This single-center, blinded, randomized, cross-over experimental study was approved by the University of Florida Institutional Animal Care and Use Committee (IACUC 202500000169) and College of Veterinary Medicine Hospital Research Review Committee (VHRRC).

### 2.2 Animals

Sample size calculations were based on an expected increase in TT from a baseline of 43 to 53°C at 30 min following SQ butorphanol administration, with an assumed standard deviation (SD) of 5 °C. Using an *⍺* of 0.006 (penalized to account for repeated measurements) and a power of 0.80, a sample size of seven horses was estimated using Stata (StataCorp, College Station, TX). To account for possible dropouts, eight adult Thoroughbred horses (four mares, four geldings) from the University of Florida research herd were included. Ages ranged from 4 to 6 years (mean ± SD 5 ± 0.8 years), and mean ± SD body weight was 557 ± 55 kg. All horses were deemed healthy based on medical history, physical examination, complete blood count, and serum biochemistry, with no evidence of chronic lameness. Horses were housed in groups of two to four under standard pasture conditions with access to grass, coastal Bermuda hay, grain twice daily, and water. On study days, horses were removed from pasture in pairs, weighed, and placed in adjacent individual stalls (4 × 4 m) bedded with wood shavings. During observation, horses maintained visual and auditory contact with their companion and had access to water; coastal hay was provided 5 h after injection. During data collection, horses were restrained with a halter and lead rope. Insect repellent was applied as needed. After each session, horses were returned to the herd.

### 2.3 Physical examination and instrumentation

A physical examination was performed after a 20-min acclimatization in the stalls. Sedation depth was assessed using the equine facial sedation scale (FaceSed) (10). Buccal mucosal color, capillary refill time (CRT), measurement of heart rate (HR) by direct auscultation, respiratory rate (*f*R) by chest excursion count, and rectal temperature were recorded. Oscillometric noninvasive blood pressure (NIBP) was measured using a multiparameter monitor (LifeWindow LW9xVet Patient Monitor; Digicare Biomedical Technology, Boynton Beach, FL, USA) with the cuff placed at the base of the unclipped tail, centered over the coccygeal artery. Cuff width was 40% of tail circumference (11). The location of the cuff was marked by clipping a line of hair adjacent to the cuff, so it could be replaced in the same position throughout the entire study period. Three measurements with less than 10% variation were averaged for systolic, diastolic, and mean arterial pressure (MAP). Values were corrected for the hydrostatic pressure difference between the tail base and right atrium (estimated at the level of the acromion) using 1 cm = 0.735 mmHg. Gastrointestinal motility was scored by abdominal auscultation of the four quadrants (0–16 scale) (4, 12). There were a total of four blinded operators performing physiologic measurements throughout the data collection period. All four operators independently performed objective measurements (e.g., heart rate, respiratory rate, mean arterial pressure), and had standardized training and measurement techniques to minimize inter-operator variability. Subjective measurements (e.g., sedation, gastrointestinal motility), by contrast, were completed by a single blinded operator (TMM) to ensure consistency of scoring. Hair was then clipped over five sites on the left side: withers, coronary band (dorsal pastern), temporomandibular joint (TMJ), 7th rib at costochondral junction, and dorsal metacarpal-phalangeal joint (D-MCP). Nostrils were not shaved. The protocol is detailed in Khatib et al. (13). After examination, horses were restrained with a halter and lead rope for TT and pain pressure threshold (PPT) testing. Thermal threshold was performed before PPT, but site testing order was randomized^1^ at the beginning of the study.

#### 2.3.1 Thermal threshold (TT)

Skin temperature at each site was recorded before stimulation using an infrared laser thermometer (Etekcity Corp., Anaheim, CA, USA). Probe baseline temperature was set to 32 °C, and the skin was warmed up with heated fluid-filled bags if <25 °C. Thermal thresholds were measured using a handheld contact heat probe (NTE-3A, Physitemp Instruments, Clifton, NJ, USA; 13 mm contact surface) with a 10-foot lead, heating ramp of 0.6 °C/s, and safety cut-off at 55 °C. The probe uses a Peltier heat pump and digital temperature control as previously described (13). Three anatomical sites were tested: the nostril, withers, and coronary band. The probe was placed on the site without pressure and held at baseline temperature for 20–30 s until panniculus muscle twitching ceased. Heating was initiated by a second operator using the remote control. Probe temperature was increased until a behavioral response occurred (panniculus muscle twitching, leg lift, lateral displacement, evasion, or head turn toward the probe) or until 55 °C. Testing was terminated remotely. Trials without response before cut-off were recorded at 55 °C.

#### 2.3.2 Pain pressure threshold (PPT)

Pain pressure thresholds were measured using a handheld pressure algometer (SMALGO, Bioseb, Vitrolles, France) with a flat 3-mm tip connected to a digital recording unit (1,000 Hz sampling, 0.2% accuracy, 0.1 g resolution). Three sites were assessed: the TMJ, the 7th rib, and the D-MCP. The device was calibrated following manufacturer instructions, before each recording. The probe was then positioned perpendicular to the skin, and force was increased at approximately 200 g/s (3 kg/cm^2^/s) until an avoidance response or the 2,500 g cut-off. Threshold was recorded automatically upon probe disconnection. The operator could view the digital display but was unaware of the pressure until stimulation ended.

For both TT and PPT, three measurements were performed at −50, −30 and −10 min prior to injection. All three values were recorded, then the average of the two closest values was used for statistical analysis. All quantitative sensory testing was performed by the same operator (TMM), blinded to treatment allocation.

### 2.4 Experimental protocol

After baseline recording, horses were randomly allocated (see text footnote 1) to receive a SQ injection above the supraspinatus muscle on the left side of the neck of either 0.1 mg/kg butorphanol tartrate (Torbugesic®, Zoetis US, Parsippany, NJ; treatment TORB) or an equivalent volume (0.01 mL/kg) of saline (treatment SAL), by the same operator (LC) not involved in the evaluations. Following a minimum one-week washout period, horses received the alternate treatment in a crossover design with those collecting data remaining blinded to treatment group. Sedation score, HR, CRT, *f*R, gastrointestinal motility score, rectal temperature and MAP were repeated at 10, 30, 45, 60, 90, 120, 180, 240, 360, and 480 min post-injection. Thermal threshold and PPT were repeated at 30, 60, 90, 120, 180, 240, 360 and 480 min post-injection. At the end of the study skin was inspected for injury, erythema or burns and horses were returned to their herd.

### 2.5 Statistical analysis

Data were recorded and processed in Microsoft® Excel for Mac, Version 16.106 (Microsoft Corporation, Kirkland, WA, USA) and analyzed using Stata/BE 17.0 for Mac (StataCorp LLC, College Station, TX, USA). Prior to analysis, TTs were also converted using the formula (threshold temperature − skin temperature)/(cutoff temperature − skin temperature) × 100 (14) and expressed as thermal excursion (TE%) for normalization. Normality of continuous variables was assessed using the Shapiro–Wilk test and visually evaluated with histograms and normal quantile (Q–Q) plots. Data were reported as mean ± standard deviation (SD) when normally distributed and as median (interquartile range, IQR) when not normally distributed. Variables normally distributed included age, body weight, HR, *f*R, MAP, rectal temperature, room temperature, TT, TE%, and PPT. FaceSed and gastrointestinal scores were rank-transformed prior to further analysis. Baseline comparisons between groups were performed using paired Student’s *t*-tests or Wilcoxon signed-rank tests, as appropriate. The effect of SQ butorphanol treatment over time was evaluated using a mixed-effects linear regression model with horse included as a random effect and time, treatment, and their interaction specified as fixed effects. For each outcome, the model took the form *Yᵢj* = *β*₀ + *β*₁(Treatmentᵢ) + *β*₂(Time*j*) + *β*₃(Treatment*ᵢ* × Time*j*) + *uᵢ* + *εᵢj*, where *Yᵢj* is the observed value for horse *i* at time *j*, *β*₀–*β*₃ are fixed-effect coefficients, *uᵢ* ~ *N*(0, *σ*^2^*ᵤ*) is the horse-specific random intercept accounting for repeated measures within horse, and *εᵢj* ~ *N*(0, *σ*^2^) is the residual error. The significance of the interaction term was assessed using a likelihood ratio test comparing nested models. Model assumptions were evaluated by inspecting residual plots. Homoscedasticity was assessed using a lowess smoother, and normality of residuals was evaluated using normal quantile plots. Post-estimation predicted marginal means were obtained using the margins command in Stata. Adjusted means for each treatment–time combination and corresponding 95% confidence intervals were derived from the fitted model. Standard errors were calculated using the delta method. Statistical significance was set at *p* ≤ 0.05.

## 3 Results

All experimental sessions began between 07:00 and 07:30 in the morning. Horses received either SAL or TORB between 08:30 and 09:40 a.m. The mean ± SD room temperature at the beginning of the first session was 14.0 ± 2.6 °C. Although room temperature was on average 2.9 °C lower at the beginning of the second session, this difference was not statistically significant (coefficient −2.9 °C, SE 1.9, 95% CI − 6.6 to 0.9, *p* = 0.14) and no significant session × time interaction was detected (LR *χ*^2^ = 5.75, *p* = 0.84). As expected, room temperature progressively increased throughout the morning, reaching statistical significance after approximately 10:00–11:00 a.m. (coefficients 4–10 °C, *p* < 0.05). Mean ± SD temperature trends over time are shown in Supplementary Figure S1.

### 3.1 Physiological variables

FaceSed score was 0 in all horses at baseline. The mixed-effects model was significant (Wald *χ*^2^ = 145.5, *p* < 0.01). A significant time × treatment interaction was detected (LR *χ*^2^ = 46, *p* < 0.01), with sedation increasing after TORB administration compared with SAL. Differences between treatments reached statistical significance at 45 min (median difference 1.3 point, SE 0.2, 95% CI 0.9–1.6, *p* < 0.01), 60 min (median difference 0.6 point, SE 0.2, 95% CI 0.3–1.0, *p* < 0.01), and 90 min (median difference 0.4 point, SE 0.2, 95% CI 0.1–0.7, *p* = 0.03). Baseline HR was 39 ± 7 bpm in the TORB treatment group and 40 ± 5 bpm in the SAL treatment group (mean difference −1.5 ± 7.5 bpm, 95% CI −7.7 to 4.7, *p* = 0.6). The model was significant overall (Wald *χ*^2^ = 55.24, *p* < 0.01). Heart rate decreased over time between 30 and 240 min after injection; however, no significant time × treatment interaction was observed (LR *χ*^2^ = 5.23, *p* = 0.9). Baseline *f*R was 20 ± 1 breaths/min in the TORB treatment group and 17 ± 2 breaths/min in the SAL treatment group (mean difference 3 ± 2 breaths/min, 95% CI −1 to 7, *p* = 0.2). A significant time × treatment interaction was detected, as *f*R slightly decreased following butorphanol administration, reaching significance at 10 min post-injection (mean difference −3 breaths/min, SE 2, 95% CI −7 to −0.1, *p* = 0.05). Respiratory rates remained within physiological limits with both treatments. Median (IQR) gastrointestinal motility did not differ at baseline between treatments (*z* = 0.5, *p* = 0.6). No significant time × treatment interaction was observed (LR *χ*^2^ = 16.1, *p* = 0.1). Baseline MAP of 93.7 ± 16.4 mmHg in the SAL treatment group was not statistically different from 100.1 ± 14.4 mmHg in the TORB treatment group (mean difference 6.3 ± 11.9 mmHg, 95% CI −3.6 to 16.2, *p* = 0.2). No significant time × treatment interaction was detected (LR *χ*^2^ = 17.3, *p* = 0.07). No horse developed hypotension. Baseline rectal temperature was 37.1 ± 2.3 °C in both groups. No significant or clinically relevant time × treatment interaction was identified (LR *χ*^2^ = 4.32, *p* = 0.9). Physiological variables are summarized in Table 1.

**Table 1:** Sedation scores and physiological variables recorded in eight adult Thoroughbred horses receiving either 0.1 mg/kg subcutaneous butorphanol tartrate (TORB) or equal volume of saline (SAL) above the supraspinatus muscle on the left side of the neck.

Variable	Treatment	Baseline	10 min	30 min	45 min	60 min	90 min	120 min	180 min	240 min	360 min	480 min
FaceSed (0–8)	TORB	0 (0, 0)	0 (0, 0.5)	0 (0, 1.5)	1 (0, 2)^b^	1 (0, 1)^b^	0 (0, 1)^b^	0 (0, 0)	0 (0, 0)	0 (0, 0)	0 (0, 0)	0 (0, 0)
	SAL	0 (0, 0)	0 (0, 0)	0 (0, 0)	0 (0, 0)	0 (0, 0)	0 (0, 0)	0 (0, 0)	0 (0, 0)	0 (0, 0)	0 (0, 0)	0 (0, 0)
HR (bpm)	TORB	39 ± 7	34 ± 3	30 ± 4^a^	35 ± 4^a^	35 ± 4^a^	33 ± 4^a^	34 ± 5^a^	34 ± 4^a^	34 ± 4^a^	37 ± 5	33 ± 5
	SAL	40 ± 5	38 ± 6	33 ± 6^a^	36 ± 5^a^	37 ± 6^a^	35 ± 4^a^	33 ± 3^a^	36 ± 2^a^	36 ± 3^a^	38 ± 5	37 ± 4
*f*R (brpm)	TORB	20 ± 4	16 ± 3^b^	16 ± 4	19 ± 5	21 ± 8	21 ± 5	24 ± 8	22 ± 4	20 ± 2	20 ± 6	20 ± 6
	SAL	17 ± 5	19 ± 4	17 ± 4	18 ± 5	17 ± 3	20 ± 7	17 ± 3	17 ± 4	20 ± 6	17 ± 3	18 ± 4
GI score (0–16)	TORB	14 (13–14)	14.5 (11.5–15.5)	14 (10–14)	12.5 (11.5–14)	13.5 (13–14)	14.5 (13–15)	14 (12.5–15)	15 (13.5–15)	14.5 (14–15.5)	16 (16–16)	15 (14.5–16)
	SAL	14 (13–15)	15 (13.5–16)	14.5 (14–14.5)	14 (12.5–15)	15 (12.5–15.5)	13.5 (11–15)	14 (13–14.5)	15.5 (14.5–16)	16 (15–16)	14 (14–16)	15 (14–16)
MAP (mmHg)	TORB	100.1 ± 14.4	92.6 ± 6.0^†^	101.3 ± 10.6	96.3 ± 7.7	95.6 ± 5.1	93.2 ± 10.6	92.4 ± 7.2	94.8 ± 10.6	96.7 ± 4.9	92.8 ± 5.8	89.3 ± 7.7
	SAL	93.7 ± 16.4	102.2 ± 13.4	95.6 ± 10.8	93.0 ± 7.8	90.1 ± 11.7	96.1 ± 10.7	91.5 ± 10.2	98.1 ± 14.9	95.9 ± 10.6	96.4 ± 14.8	94.2 ± 6.5
Rectal T° (°C)	TORB	37.1 ± 0.2	37.3 ± 0.5	37.2 ± 0.6	37.4 ± 0.5	37.4 ± 0.5	37.3 ± 0.7	37.4 ± 0.6	37.4 ± 0.4	37.6 ± 0.4	37.5 ± 0.2	37.5 ± 0.3
	SAL	37.1 ± 0.3	37.4 ± 0.4	37.4 ± 0.3	37.5 ± 0.3	37.5 ± 0.3	37.5 ± 0.3	37.5 ± 0.4	37.4 ± 0.4	37.4 ± 0.3	37.5 ± 0.3	37.5 ± 0.3

a Indicates statistically significantly different from baseline (*p* ≤ 0.05).

b Indicates statistically significantly different between treatments (*p* ≤ 0.05).

FaceSed: equine facial sedation scale; *f*R, respiratory rate; GI, gastrointestinal; HR, heart rate; MAP, mean arterial pressure.

### 3.2 Thermal threshold (TT)

At the coronary band, baseline TT was significantly higher in the TORB (50.0 ± 4.7 °C) than in the SAL treatment group (38.9 ± 5.6 °C; mean difference 11.4 ± 7.7 °C, 95% CI 4.9–17.1, *p* = 0.004). Model comparison using likelihood ratio tests demonstrated a significant treatment × time interaction (LR *χ*^2^ = 18.1, *p* = 0.02). Between 60 and 240 min post-injection, TT was 2–5.5 °C lower in the TORB treatment group relative to SAL. Similarly, although TE% was higher at baseline in the TORB treatment group (coefficient 35.8%, SE 13.6%, 95% CI 9.3–62.4, *p* = 0.008), a significant negative treatment × time interaction was observed (LR *χ*^2^ = 18.4, *p* = 0.02), with TE% 8.6–26.6% lower in the TORB treatment group compared with SAL treatment group between 60 and 240 min.

At the withers, baseline TT was 42.2 ± 6.2 °C in the TORB treatment group and 42.7 ± 6.3 °C in the SAL treatment group (*p* = 0.87). Baseline TE% was 48.1 ± 23.2% and 54.2 ± 21.2%, respectively. No significant differences between treatments over time were observed for TT (Wald *χ*^2^ = 11.32, *p* = 0.83) or TE% (Wald *χ*^2^ = 15.57, *p* = 0.55).

At the nostrils, baseline TT was 39.4 ± 2.7 °C in the TORB group and 38.3 ± 2.7 °C in the SAL group (*p* = 0.5). Baseline TE% was 34 ± 12.6% and 32 ± 15.6% in the TORB and SAL groups, respectively (*p* = 0.8). No significant treatment × time interaction was detected for TT (Wald *χ*^2^ = 12.34, *p* = 0.78) or TE% (Wald *χ*^2^ = 7.59, *p* = 0.97) at the nostrils. Thermal threshold curves are represented in Figure 1.

**Figure 1:**
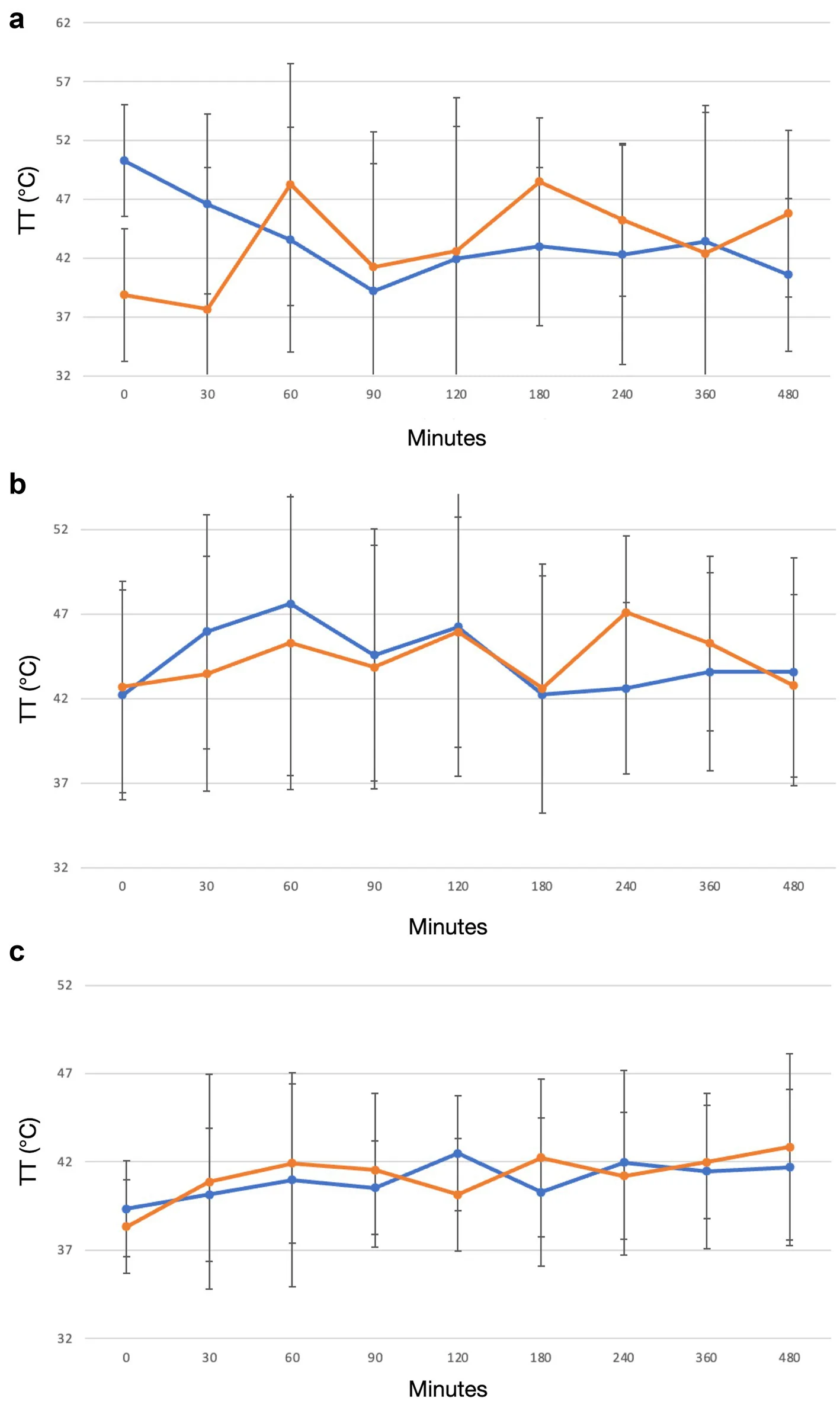
Representation of mean ± SD thermal threshold (TT; expressed in °C) over time recorded at the **(a)** coronary band, **(b)** withers and **(c)** nostrils in eight healthy Thoroughbred horses receiving either 0.1 mg/kg subcutaneous butorphanol tartrate (blue) or equal volume of saline (orange) above the supraspinatus muscle on the left side of the neck.

### 3.3 Pain pressure threshold (PPT)

At the 7th rib, baseline mean ± SD PPT was 1389.8 ± 495 g in the TORB treatment group and 1245.2 ± 416.0 g in the SAL treatment group. The mean difference of 144.6 ± 678.9 g was not statistically significant (*t* = 0.6, *p* = 0.57). Although PPT slightly increased starting at 90 min after butorphanol administration, no significant treatment × time interaction was detected (LR *χ*^2^ = 11.44, *p* = 0.18). The study was adequately powered to detect the difference at 180 min, when the largest mean difference (644.6 g, SE 278, 95% CI 99.7–1189.5 g) was observed. However, given the large PPT variability, at least 11 horses would have been required to detect a difference at 90 or 120 min.

At the D-MCP joint, baseline mean ± SD PPT was 1213.7 ± 806.8 g in the TORB treatment group and 1934.9 ± 1191.1 g in the SAL treatment group, corresponding to a mean difference of 721.2 ± 811.9 g (*t* = −2.5, *p* = 0.04). The mixed-effects model was significant overall (Wald *χ*^2^ = 42.18, *p* < 0.001). Despite the baseline difference between groups, no significant change in PPT over time or treatment × time interaction was detected. Substantial inter-individual variability was observed. A modest increase in PPT following butorphanol administration was noted, peaking at 60 min; however, to detect an increase of the observed 807 g with an SD of the mean difference of about 1,000 g, an estimated sample size of 15 horses would have been required.

At the TMJ, baseline mean ± SD PPT did not differ between TORB (519.6 ± 410.3 g) and SAL (670.0 ± 365.5 g) treatments (mean difference 150 ± 519 g, 95% CI − 585.0 to 284.3, *p* = 0.44). No significant differences in PPT were observed between treatments over time (Wald *χ*^2^ = 17.29, *p* = 0.44). PPT curves are represented in Figure 2.

**Figure 2:**
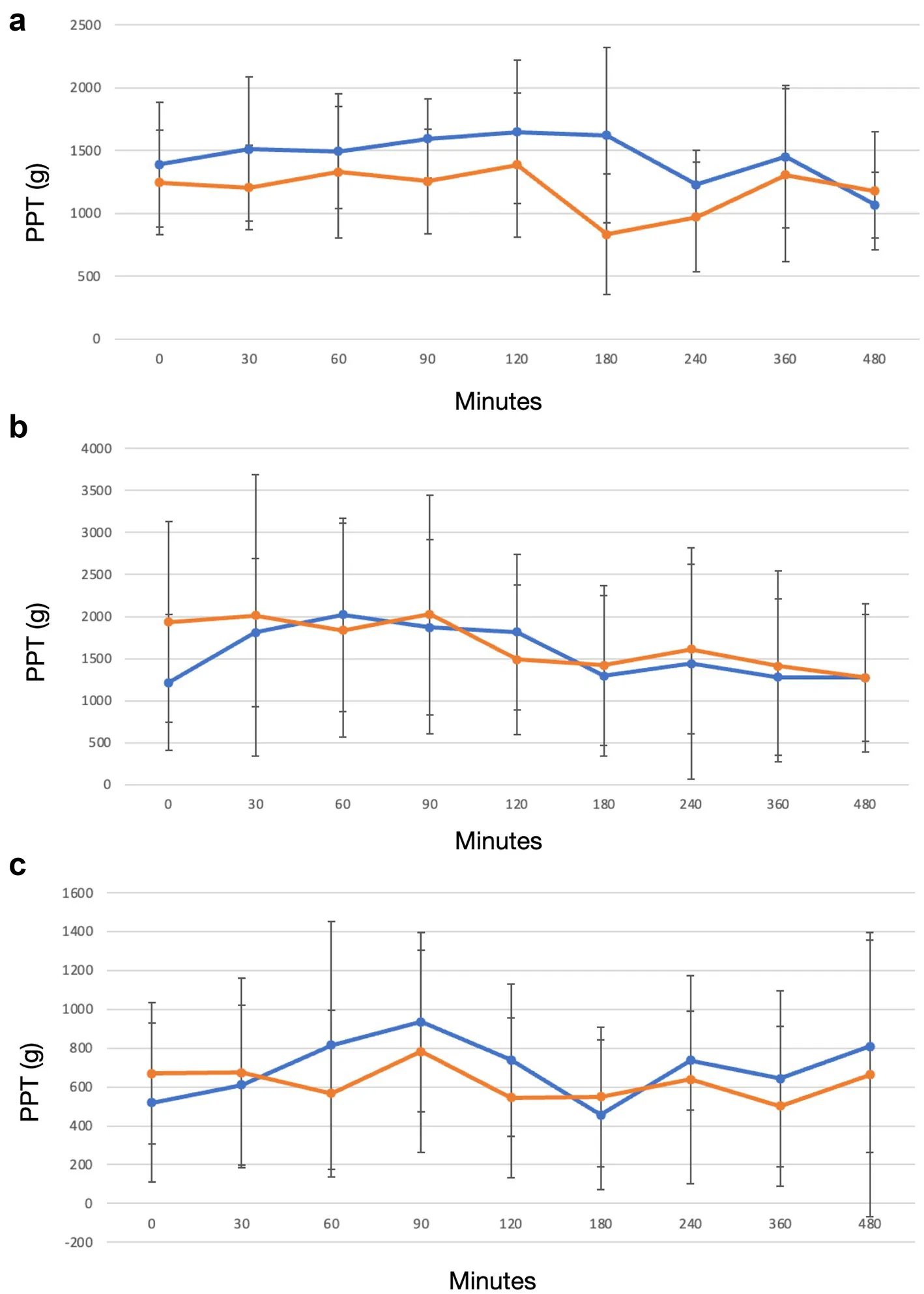
Representation of mean ± SD pain pressure threshold (PPT; expressed in g) over time recorded at **(a)** 7th rib, **(b)** dorsal metacarpal-phalangeal joint and **(c)** temporomandibular joint in eight healthy Thoroughbred horses receiving either 0.1 mg/kg subcutaneous butorphanol tartrate (blue) or equal volume of saline (orange) above the supraspinatus muscle on the left side of the neck.

### 3.4 Adverse effects

No excoriations, edema, erythema, or burns were observed at any of the testing sites. Six horses developed a localized sweat patch at the site of injection after SQ butorphanol, that resolved within a couple of hours (Figure 3).

**Figure 3:**
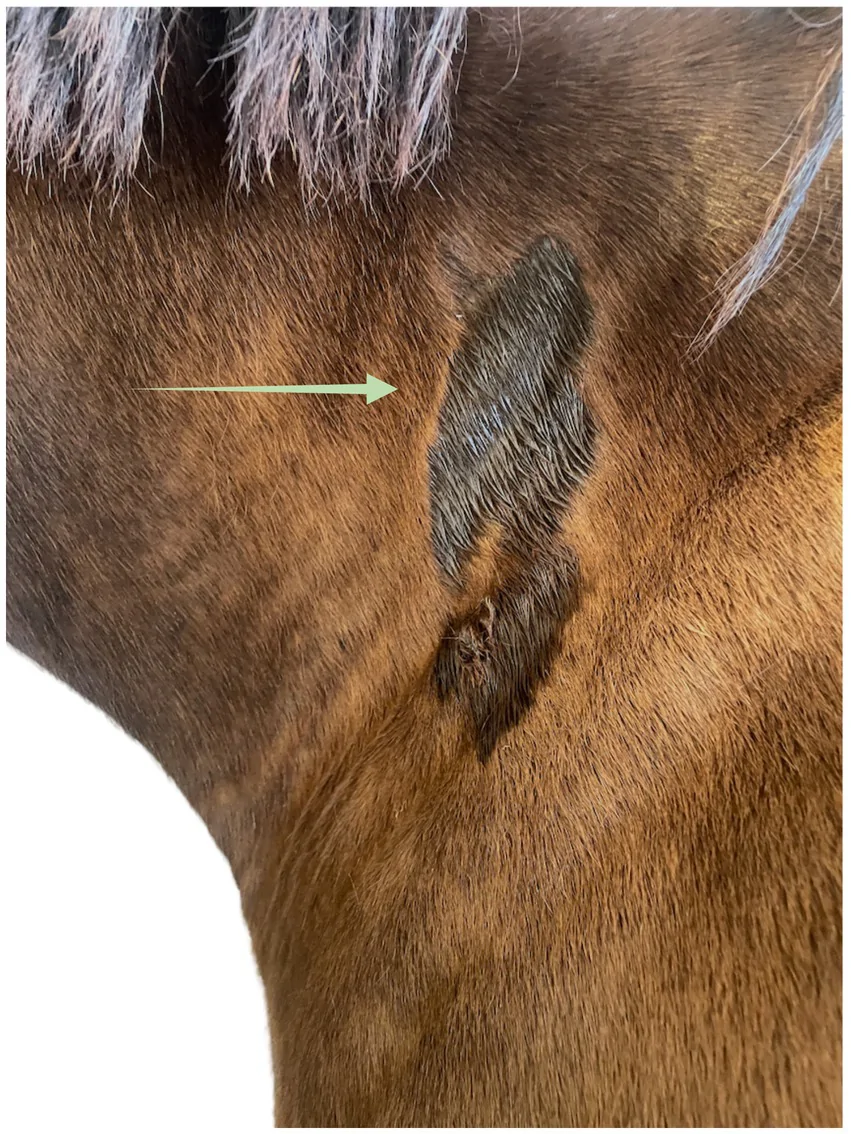
Localized sweating observed at the injection site on the neck following subcutaneous administration of 0.1 mg/kg of butorphanol tartrate. The affected area appears as a discrete, linear patch of damp, darkened hair. This focal sudomotor response resolved spontaneously within approximately 2 h, with no associated systemic signs.

## 4 Discussion

The present study demonstrated limited clinical analgesic efficacy of SQ butorphanol when assessed using multiple quantitative sensory testing modalities in healthy adult horses. These findings are consistent with previous work (3, 5, 7, 15) demonstrating mild and transient analgesic efficacy of butorphanol in horses at clinically relevant doses. The apparent decrease in TT at the coronary band between 60 and 240 min post-injection likely reflects normalization from an elevated baseline, or it was potentially due to behavioral artifact such as subtle weight-shifting during probe application, rather than a genuine reduction in nociceptive threshold. In contrast, PPT was highly variable across individuals, and no consistent treatment effect was detected at any of the three sites tested.

Receptor density/distribution differ between visceral and somatic afferents. Specifically, peripheral κ-opioid receptors are considered to be primarily involved in the modulation of visceral rather than somatic nociception (16), which has been used to support the clinical use of butorphanol for visceral pain conditions such as colic. However, visceral pain is referred to corresponding somatic dermatomes and consciously perceived as somatic pain. Models of cecal (17), duodenal (5, 18, 19) and colorectal (20) dilation have previously been used to simulate visceral nociception more directly but were considered too invasive for the present study design. Stimulation at the seventh costochondral junction was instead selected to approximate the projected dermatome of cranial abdominal pain and did not demonstrate any increase in PPT following butorphanol administration. Even if we were testing visceral pain through its somatic dermatome projection, the findings are broadly consistent with distension-model studies recording mild and inconsistent analgesic effects (5, 17, 20). Notably, other studies using comparable or higher intravenous doses of butorphanol (6, 15) also reported only brief or inconsistent somatic antinociception, arguing against underdosing as the primary explanation and suggesting instead a pharmacologic ceiling on butorphanol’s somatic antinociceptive efficacy, independent of dose. This clinically irrelevant antinociceptive results may partly reflect an overlap between antinociceptive and sedative drug actions. Indeed, SQ butorphanol produced mild, transient, but statistically significant sedation, peaking between 45 and 90 min post-injection, with minimal physiological impact, consistent with previous reports (1). While sedation was statistically significant, its clinical magnitude was mild: the maximum FaceSed score recorded was 3/18 (in a single horse at a single timepoint), well below the threshold (>5/18) identified by (10) as necessary for adequate sedation during standing procedures. This suggests sedation depth was likely insufficient to meaningfully blunt behavioral responsiveness to the thermal or mechanical stimuli, though we cannot fully exclude a subtle masking effect given the absence of a validated method for quantifying the sedation/nociception interaction independent of the behavioral endpoints themselves. Physiological findings were also in agreement with a previous study using a comparable SQ dose (4), with mild and transient reductions in HR and fR; unlike that study, however, no significant reduction in gastrointestinal motility score was observed here.

An observation not previously reported, to the authors’ knowledge, was the presence of a localized sweat patch at the injection site in the majority of horses following SQ butorphanol but not following saline administration. No other signs of inflammation were observed at the injection site in any horse. Localized swelling, mild inflammation, local sweating, and edema have similarly been reported following SQ meperidine (21) and SQ ketamine (22) injections in healthy horses, suggesting a reaction pattern not unique to butorphanol. Benzethonium chloride, a preservative common to the commercial formulations of both butorphanol and ketamine, is a plausible shared contributor. It is structurally related to benzalkonium chloride (BAC), a well-documented moderate irritant in Draize, patch-test, and murine ear-swelling assays and showed cross-reactivity with BAC in skin testing (23). However, the commercial meperidine formulation used in (21) contained no such preservative, relying instead on an acetate buffer; so benzethonium chloride alone cannot account for the shared reaction across all three drugs. Another possible explanation may be the acidic pH common to all three formulations (meperidine ~3.5–6.0, ketamine and butorphanol ~3.5–5.5), which could directly irritate subcutaneous tissue. An acidic, hypertonic, or otherwise chemically irritating bolus in the subcutaneous space can activate local nociceptors, triggering an axon-reflex flare and, in horses specifically, localized sudomotor activation via sympathetic discharge. The precise mechanism remains speculative and warrants further investigation, ideally through direct comparison of preserved versus preservative-free and pH-matched versus unmatched formulations.

Several limitations should be acknowledged. Thermal threshold and PPT testing are well-validated methodologies for objectively assessing nociception and analgesic efficacy in horses, making them ideal for evaluating the effectiveness of pharmaceuticals in research settings (8, 24–27). However, horses in this study had limited prior habituation to the testing devices, and anticipatory or anxiety-related responses at previously tested sites cannot be excluded. The study population was also fairly homogeneous with respect to breed, sex, and age; while these variables have not previously been shown to substantially influence opioid analgesic response, the resulting lack of variability may limit generalizability to the broader clinical population. Randomization to treatment order did not fully eliminate baseline differences between treatment groups at some sites (e.g., PPT at the D-MCP joint, TT at the coronary band); although these differences were accounted for by the mixed-effects modelling approach, they may partly reflect the small sample size (*n* = 8) rather than a true treatment-independent effect. Because the mixed-effects models estimate the treatment × time interaction as each horse’s change from its own baseline rather than as a fixed between-group comparison at a single timepoint, baseline imbalance at these sites does not, by itself, bias the interaction estimates; it would only do so if the imbalance were systematically related to each horse’s rate of change over time, for which there was no evidence in the present dataset. The lack of a temperature-controlled indoor research facility resulted in variable ambient temperature during testing. The relationship between environmental temperature and nociceptive threshold is complex, and the literature reports conflicting findings across species. To help account for this, TT was expressed as thermal excursion percentage (TE%) to make a more accurate representation of change between testing time points. However, this normalization does not fully resolve the underlying confound, as reduced tissue temperature slows peripheral nerve conduction velocity and thereby shifts the biological response threshold itself, rather than merely rescaling the recorded temperature. Sites with skin temperature below 25 °C were partially compensated for by pre-warming to 30–32 °C, and the thermode baseline was standardized at 32 °C, consistent with the DFNS protocol used in human QST research (28). Finally, this study was conducted in healthy, pain-free horses; the antinociceptive response to SQ butorphanol observed here may not directly extrapolate to horses experiencing acute visceral pain, such as colic, in which altered peripheral and central pain processing (e.g., sensitization) could modify drug efficacy.

## 5 Conclusion

Collectively, these results suggest that 0.1 mg/kg SQ butorphanol provides limited antinociception in healthy, pain-free horses under the experimental conditions tested. Subcutaneous butorphanol should not be relied upon as a consistent antinociceptive treatment in adult horses, and its clinical use, including for analgesia prior to referral transport in colic cases, should be regarded as adjunctive rather than as a substitute for other validated analgesic strategies.

## Data Availability

The raw data supporting the conclusions of this article will be made available by the authors, without undue reservation.

## Footnotes

1
www.randomizer.org
