# Supplementary material for: Antinociceptive effects of subcutaneous butorphanol administration in healthy horses
Source: Front Vet Sci. 2026 Oct 9;13:1970768. doi: 10.3389/fvets.2026.1970768 (PMC13691309; doi:10.3389/fvets.2026.1970768)
Supplement: Supplementary file 1 [file Image_1.pdf]

Supplementary Material

1 Supplementary Figures

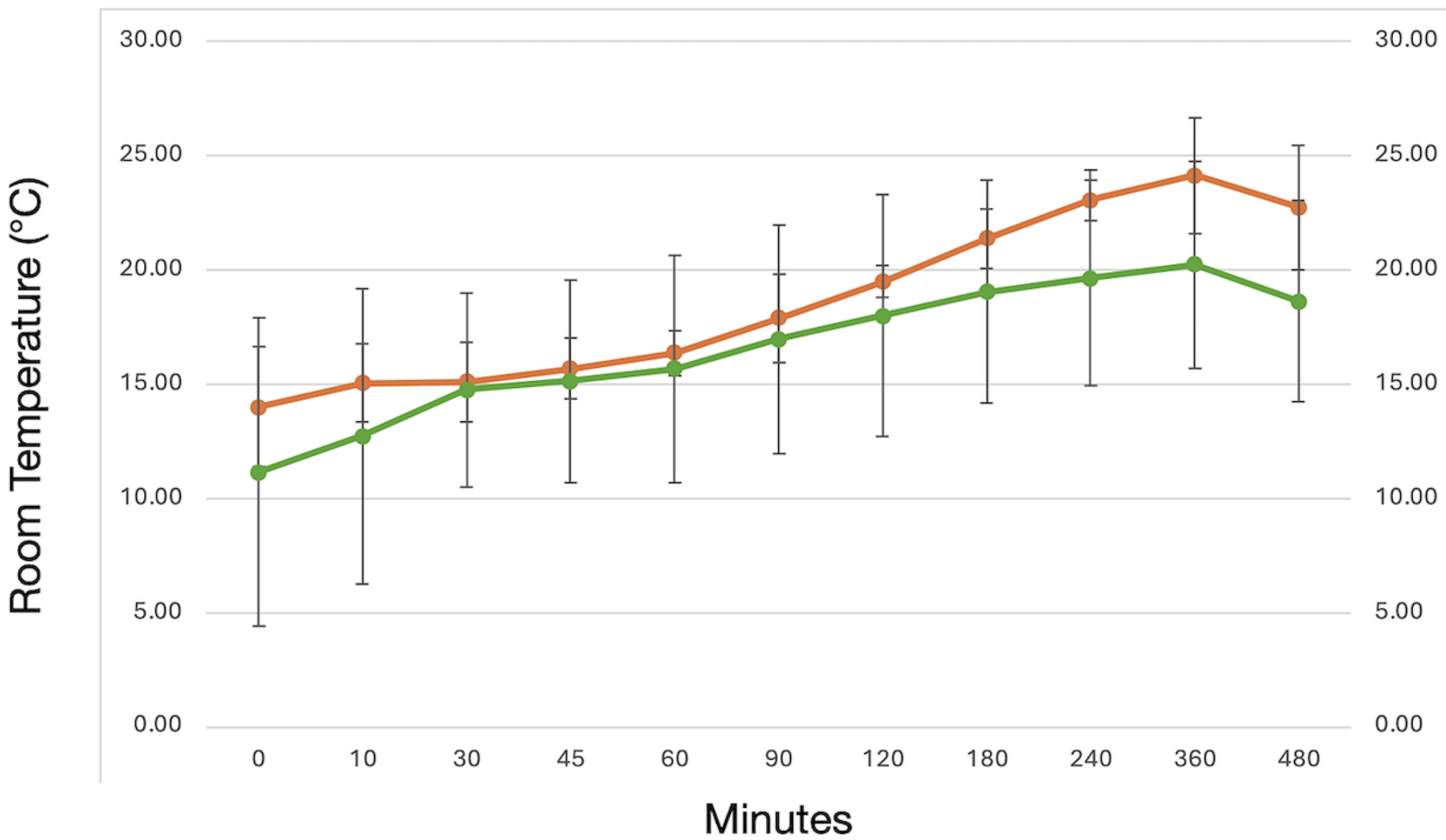

Supplementary Figure 1. Representation of mean ± SD recorded ambient temperatures **(**expressed in °C), during the first (orange) and second (green) session of the experiment.
